# Transcriptome classification reveals molecular subtypes in psoriasis

**DOI:** 10.1186/1471-2164-13-472

**Published:** 2012-09-12

**Authors:** Chrysanthi Ainali, Najl Valeyev, Gayathri Perera, Andrew Williams, Johann E Gudjonsson, Christos A Ouzounis, Frank O Nestle, Sophia Tsoka

**Affiliations:** 1 Centre for Bioinformatics, Department of Informatics, School of Natural and Mathematical Sciences, King’s College London, Strand, London, WC2R 2LS, UK; 2 St John’s Institute of Dermatology, Division of Genetics and Molecular Medicine, King’s College London, Tower Wing, Guy’s Hospital, Great Maze Pond, London, SE1 9RT, UK; 3 Centre for Systems, Dynamics and Control, College of Engineering, Mathematics and Physical Science, University of Exeter, Exeter, EX4 4QF, UK; 4 Department of Dermatology, School of Medicine, University of Michigan, Box 0932, Ann Arbor, MI 48109-0932, USA; 5 Present address: Computational Genomics Unit, Institute of Agrobiotechnology, Centre for Research & Technology Hellas, Thessaloniki, Greece; 6 Present address: Donnelly Centre for Cellular & Biomolecular Research, University of Toronto, 160 College Street, Toronto, ON, M5S 3E1, Canada

**Keywords:** Disease classification, Molecular grouping, Psoriasis, Decision tree prediction model

## Abstract

**Background:**

Psoriasis is an immune-mediated disease characterised by chronically elevated pro-inflammatory cytokine levels, leading to aberrant keratinocyte proliferation and differentiation. Although certain clinical phenotypes, such as plaque psoriasis, are well defined, it is currently unclear whether there are molecular subtypes that might impact on prognosis or treatment outcomes.

**Results:**

We present a pipeline for patient stratification through a comprehensive analysis of gene expression in paired lesional and non-lesional psoriatic tissue samples, compared with controls, to establish differences in RNA expression patterns across all tissue types. Ensembles of decision tree predictors were employed to cluster psoriatic samples on the basis of gene expression patterns and reveal gene expression signatures that best discriminate molecular disease subtypes. This multi-stage procedure was applied to several published psoriasis studies and a comparison of gene expression patterns across datasets was performed.

**Conclusion:**

Overall, classification of psoriasis gene expression patterns revealed distinct molecular sub-groups within the clinical phenotype of plaque psoriasis. Enrichment for TGFb and ErbB signaling pathways, noted in one of the two psoriasis subgroups, suggested that this group may be more amenable to therapies targeting these pathways. Our study highlights the potential biological relevance of using ensemble decision tree predictors to determine molecular disease subtypes, in what may initially appear to be a homogenous clinical group. The R code used in this paper is available upon request.

## Background

Psoriasis is one of the most prevalent chronic inflammatory disorders caused by an interplay of genetic factors and the environment on the background of dysregulated immune system [1]. The disease affects 2 - 3% of the population worldwide [2] and can be variable in morphology, severity and distribution. There are several clinical variants of psoriasis, but the most common variant, plaque psoriasis, is characterised by chronic, symmetrical, silvery-scaled, sharply circumscribed plaques [1,3]. Plaque psoriasis is the most common form of the disease and can begin in childhood and late adolescence (Type 1) or in adulthood (Type 2), with a predilection for elbows, knees and the scalp.

Although the cause of psoriasis remains unknown, it is thought to be a complex and multifactorial disorder brought about by the combination of multiple susceptibility genes [4-6], a dysregulated immune system [7,8] and environmental factors [9]. Through Genome Wide Association Studies (GWAS) [10,11], a number of genetic variants have been identified as contributing towards psoriasis pathogenesis. A unifying model that integrates genetic, environmental and immunological aspects of skin inflammation has been proposed [12].

In recent years, progress has been made in understanding the pathogenesis and treatment of psoriasis. Pathogenesis is mainly linked to activation of several types of leukocytes that control cellular immunity and to a T-cell-dependent inflammatory process in skin that accelerates the growth of epidermal and vascular cells in psoriasis lesions. Current therapeutic approaches against the disease take advantage of proteins or antibodies aiming either at specific inflammatory co-activators or more generally at immune cells [3]. While there is now increasing insight into the genes conferring disease susceptibility, much less is known about the types of regulatory networks of expressed genes which define the molecular signature of the disease.

The first large-scale and detailed gene expression studies of psoriasis identified various differentially expressed genes by comparing non-lesional and lesional skin against normal tissue [13-16]. Recent studies have attempted to elucidate the molecular pathways underlying in psoriasis [17-20]. However, determining genes that contribute to complex human disorders through analysis of microarray data is challenging due to the large number of gene predictors, their possible interactions, and the small number of samples. Termed the “small n, large p” problem [21], this implies that classical statistical methods cannot be implemented directly in functional genomics approaches for the identification of diagnostic or prognostic biomarkers. In this respect, decision trees have proven to be a sensible non-parametric method for classification and variable selection [22]. Random forest (RF) classification is an ensemble of CART decision trees and has been found to outperform other machine learning techniques for analysis of microarray data [23-26].

In this study, a computational methodology based on decision tree predictors is developed to discover molecular sub-groups from gene expression data and illustrate gene signatures associated with each group. The random forest (RF) algorithm [22] is used here to (i) cluster psoriasis transcripts into subgroups and (ii) discriminate between disease phenotypes and generate gene signatures that best differentiate them. RF has been shown to be robust in noisy data, to avoid over-fitting in cases where the number of features is larger than the number of observations and to be particularly suitable for the feature selection process [24,27,28].

More specific to the current analysis, we first analysed gene expression profiles in normal and disease skin tissues, so as to define common differentially expressed genes. This core gene set was then used to group psoriatic tissue samples through RF clustering of real and synthetic data, as previously developed [29]. This step resulted in dividing psoriatic tissues into two subgroups according to similarity of gene expression patterns. Finally, RF classification was used to derive gene signatures able to discriminate between normal and disease phenotypes, including the above-proposed new psoriatic subgroups. Such gene signatures are discussed in following sections with respect to their effect on defining distinct molecular characteristics and were validated through comparisons with other psoriasis gene expression studies.

Molecular profiling of psoriatic phenotypes followed by classification of tissue samples into appropriate disease classes has the potential to derive clusters of similar transcription responses from the entire repertoire of profiles generated. Especially in the case of a homogeneous clinical patient group, such as plaque-type psoriasis, the classification of transcriptional patterns into appropriate sub-groups may reveal distinct molecular mechanisms that may operate within this group and may explain variability in response and options of disease treatment. Overall, given the predictive nature of the decision model employed, such patient categorisations can lead to significant insights into disease mechanisms and novel, targeted therapeutic approaches.

## Results and Discussion

**Figure 1:**
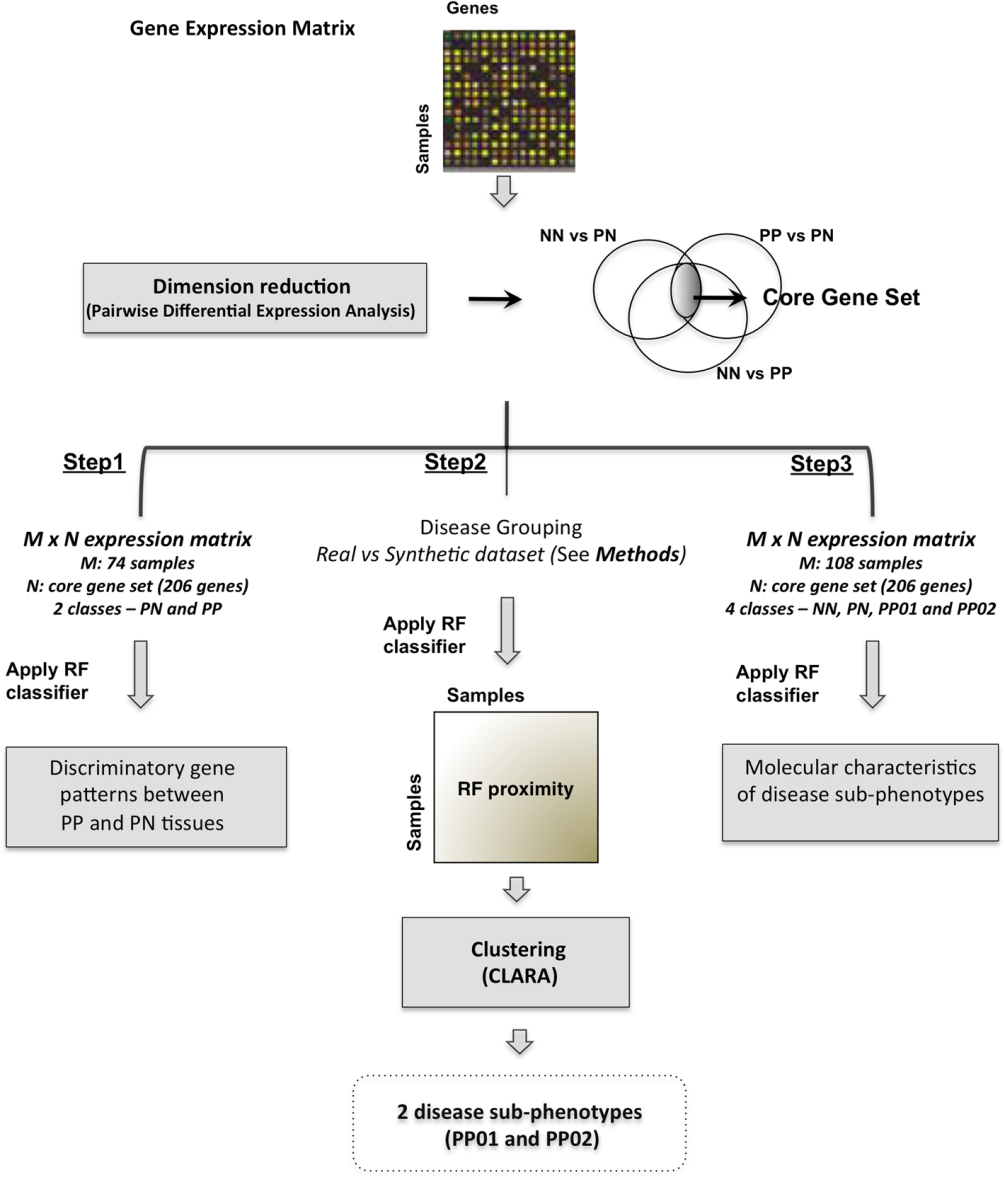
**Pipeline for patient stratification.** For further details on related methodology, please see main text.

A pipeline (Figure 1) for patient stratification according to gene expression profiles in psoriasis was implemented to generate molecular sub-groups and uncover gene signatures associated with each disease group. Such an approach has possible predictive implications: given relevant expression measurements for key signature genes, uncharacterised tissue samples can be ascribed to these predefined disease classes, which can reflect different disease prognosis or response to treatment.

### Gene expression patterns define a core set of dysregulated genes among normal, non-lesional and lesional skin

**Figure 2:**
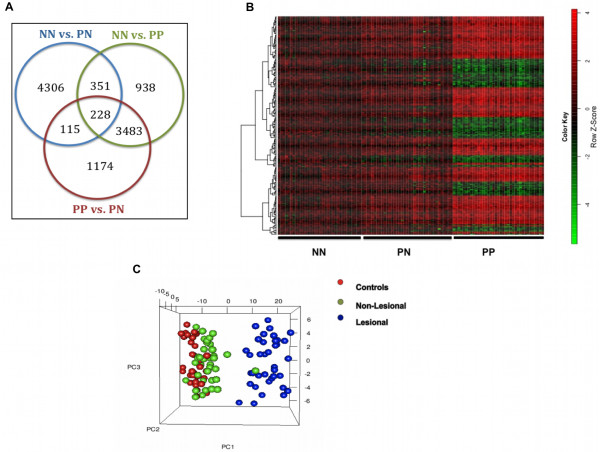
**Differential gene expression in lesional (PP), non-lesional (PN) and normal (NN) skin tissue.** Gene expression was analysed to reveal probe sets that were differentially expressed between pairwise comparisons of PP, PN and NN tissue groups. (**A**) The Venn diagram shows the number of probe sets identified in each of the three differential analyses performed. Probe sets common to all three pairwise comparisons were 228 (206 genes). (**B**) Microarray analysis of 108 skin tissue samples (in columns) for 206 genes (in rows) common to all tissue types, identified through differential expression analysis. Tissues have been grouped according to disease phenotype (normal NN, non-lesional PN and lesional PP) and heatmap colours indicate z-score of each gene expression value against the mean of corresponding normal values, (green: decreased expression, red: increased expression, inset). Similarity of gene expression vectors across all samples is represented by the dendrogram on the left. (**C**) Principal Component Analysis to suggest sample clustering across skin types according to gene expression patterns. Good separation of inflamed (PP) and non-inflamed (PN, NN) tissues was observed, indicating a progression from normal (red) to lesional skin (blue) through the non-lesional cases (green).

Skin samples from psoriatic patients were either of inflamed, lesional type (PP, involved skin) or non-inflamed, non-lesional tissue (PN, uninvolved skin). These were analysed together with skin samples from normal individuals (denoted as NN). Relevant gene expression measurements for the disease classes (NN, PN and PP) were obtained through the Genetic Association Information Network (GAIN, see Methods). Differential expression analysis was performed and involved three pairwise comparisons between tissue datasets (i.e. NN vs. PN, PN vs. PP and NN vs. PP), resulting in three sets of differentially expressed probe sets per pair (p-value < 0.05 and FDR < 0.05). A set of 228 probes was shared across all datasets (Figures 1 and 2a) and corresponded to a total number of 206 unique genes, of which 130 genes were over-expressed and 76 under-expressed in PP samples compared to NN (Additional file 1: Table S1). This group of genes constituted a *core* set of genes expressed differentially across the three disease phenotypes and was used to derive disease-specific expression patterns in the RF-based procedure described in the following sections.

Unsupervised hierarchical clustering was carried out on the set of 206 core genes to explore and visualise the patterns of gene expression from normal (NN) to non-lesional (PN) and then to lesional (PP) skin samples. Figure 2b shows an overview of gene expression for the core probe sets, clustered according to similarity of expression across NN, PN and PP samples. This visualisation represents a striking outline of the varying transcriptional patterns at each disease phase, progressing gradually from generally non-differentiated gene expression in non-inflamed tissues (NN, PN), to markedly differentiated genes in lesional samples (PP).

Principal component analysis (PCA) was used to assess the clustering of samples when progressing from un-inflamed to inflamed skin. There was a clear distinction between lesional (PP) and non-lesional (NN and PN) phenotypes (Figure 2c), manifested as distinct clusters of samples from normal to the involved phenotype through non-involved skin. Normal and psoriatic un-involved samples (NN and PN) co-clustered away from involved cases (PP), in agreement with previously published analyses [16,19]. This demonstrated the changes in gene expression profiles across NN, PN and PP skin and revealed a marked difference between inflamed (PP) skin and un-inflamed (PN and NN) phenotypes.

Among the strongly dysregulated genes in the core gene set (Additional file 1: Table S1), several of the under-expressed genes were found to encode proteins involved in fibrotic processes and immune responses. For example, *FN1*, *PDGFC*, *MYH10* are involved in the regulation of the actin cytoskeleton, which participates in fundamental processes such as the regulation of cell shape, motility and adhesion [30]. *DIXDC1*, *CGNL1 and* SSPN encode cell adhesion and junction proteins. Betacellulin (*BTC*), *IL1F7*, *CD81*, *FN1*, *PDGFC* and *SCARB2* are immune response genes. In addition, *MEGF9*, *BTC*, *FN1*, *PHF2* belong to the family of growth factors that activate the epidermal growth factor receptor, *EGFR* (*ErbB1*) and according to a previous study *BTC* plays an important role in skin morphogenesis [31]. Among the over-expressed genes, several participate in keratinocyte proliferation and differentiation (*EREG*, *KLK8* and *PPARD*). Of note is *KLK8*, potentially involved in the modulation of hyperkeratosis in a psoriatic lesion and may be implicated in preventing excessive keratinocyte proliferation, resulting in increased shedding of corneocytes. This is clinically reflected in the copious quantities of scale that are shed by psoriasis patients [32]. Genes *LTB4R2* and *PPARD* are also involved in keratinocyte migration. Finally, a group of up-regulated genes *SNRPG*, *SNRPD1*, *SNRPD3*, *SNRPA1*, *SNRPC*, *SF3B14*, *SFRS9* is involved in spliceosomal assembly. Overall, most dys-regulated genes were found to be consistent with current knowledge.

### Distinctive gene expression patterns between lesional and non-lesional tissues (PP vs. PN)

Following the general patterns of psoriatic tissue differentiation, the use of decision tree ensembles was explored to classify samples into PN and PP classes and derive the major gene patterns able to discriminate the psoriatic phenotypes (see Figure 1, *step1*). We used 74 tissue samples from psoriasis patients, each characterised by a vector of core gene expression values, and a random forest (RF) classifier [22] was applied to distinguish samples in lesional (PP) and non-lesional (PN) phenotypes. The classifier employed 1000 trees with training of each tree performed on 2/3 of samples and testing on the remaining 1/3 (see Methods and Additional file 2: Supplementary Methods). The prediction accuracy of the classifier was high (accuracy 97.3%, OOB error rate 2.7%).

**Figure 3:**
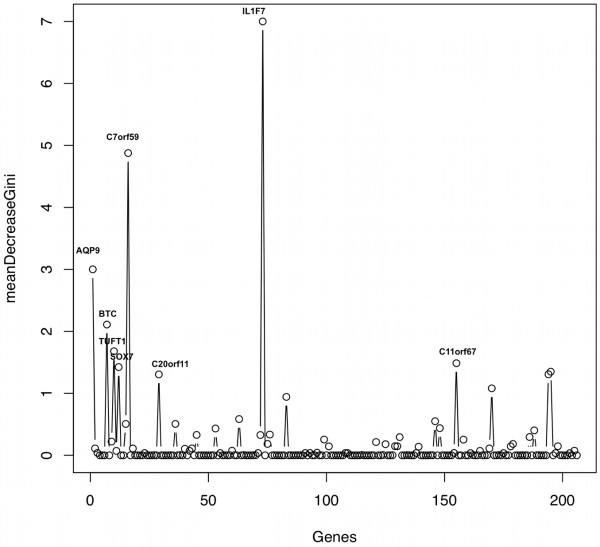
**Informative genes for the classification of skin samples in lesional and non-lesional classes (PP and PN, respectively).** Gini Index (GI) was used to generate a variable importance measure and provide an estimate of feature (gene) relevance to disease state. The five most important genes in determining disease classes were *IL1F7*, *C7orf59*, *AQP9*, *BTC* and *TUFT1.*

The random forest classifier was then used to indicate the relevant importance of features in the classification, i.e. which genes are more important in predicting the appropriate disease class. Genes were ranked through the Gini Index (GI) in terms of their discriminative power (see Methods) and Figure 3 shows genes with the highest GI when distinguishing inflamed (PP) from non-inflamed (PN) skin. Five genes indicated through this procedure were *IL1F7*, *C7orf59*, *AQP9*, *BTC* and *TUFT1* and were all related to immune response processes.

### Identification of molecular sub-types within psoriatic tissue samples

In addition to key patterns that defined disease outcome in psoriatic tissues above, we used random forest in unsupervised mode, as a clustering platform to group lesional psoriasis samples based on their gene expression properties (see Figure 1, *step 2*). The aim was to generate two sub-groups among disease tissues (PP), before further classification runs could identify molecular differences among them (Figure 1, *step 3*, discussed later). As described previously [29], first synthetic data were generated by randomly sampling the gene expression observations. Then, a random forest predictor was built to distinguish the real from synthetic data (see Methods) and define a similarity measure between the psoriatic cases in the form of the random forest proximity measure. Finally, CLARA clustering of the proximity matrix partitioned the psoriatic cases into two groups, named PP01 and PP02 (Figure 1, *step 2*). The adjusted rand index to indicate the difference between the two identified sub-groups was -0.0269.

The RF-derived proximity measure can be used to generate a multi-dimensional scaling (MDS) plot, where dissimilarities between samples return a set of points in low dimensional Euclidian space, similar to principal component analysis. The MDS plot projects data into a 2D space giving the similarities among patients and their respective classes. The distinction of samples in two groups, PP01 (red circles) and PP02 (black circles) is shown through the MDS plot in Additional file 3: Figure S1. Similar clustering of PP phenotypes in two clusters has been noted through hierarchical clustering (data not shown) and was used as means of determining the optimum number of psoriatic sub-groups.

The relationship between these sub-groups and clinically measurable parameters, was assessed. Psoriasis Area and Severity Index (PASI), Body Mass index (BMI), Age of Onset, Age and Body Surface Area (BSA) were evaluated against subgroups PP01 and PP02. Of these, age was found to be significantly altered between the two subgroups (p-value 0.0184, Wilcoxon signed-rank test). It is emphasised here that plaque-type psoriasis constitutes a homogeneous clinical group, distinct from other forms of psoriasis. Therefore, it is not surprising that such coarse-grained clinical parameters can not capture the subtle differences in plaque psoriasis sub-groups (PP01, PP02). Instead, our focus here is to distinguish the underlying biological mechanisms, in terms of distinct biochemical pathways and interactions that act in these subgroups, as we report in following sections.

**Figure 4:**
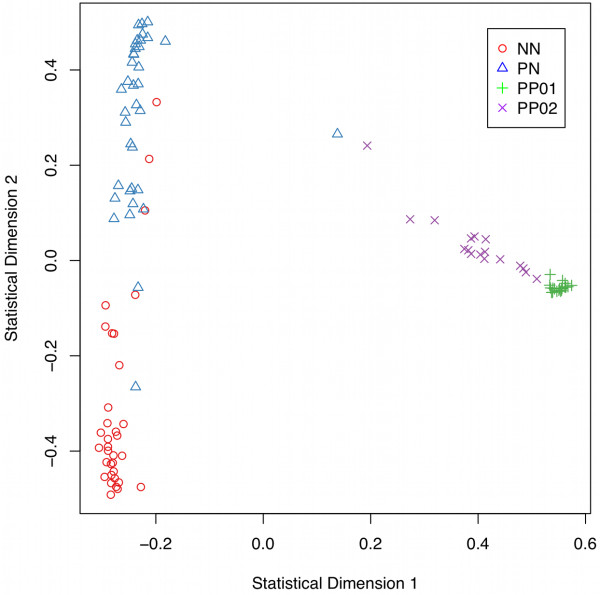
**A multidimensional scaling (MDS) plot to illustrate the molecular grouping of samples.** A dissimilarity matrix in random forest is constructed through the use of synthetic data drawn from the distribution of psoriatic samples (see Methods). Patients are clustered according to these dissimilarities and two distinct psoriatic groups are identified, PP01 (green) and PP02 (purple). All lesional samples (PP) cluster away from both normal (NN) and non-lesional (PN) tissue samples, in accordance to observations in figure 2c.

Having separated psoriatic samples in two sub-groups, a new gene expression matrix, where PP samples were split in PP01 and PP02, was used as input to another round of RF classification (see Figure 1, *step 3*). The core genes (total of 206) were used as variables to classify 108 samples in any of the four classes (normal NN, non-lesional PN, first lesional group PP01, or second lesional group PP02). The purpose of this series of experiments was to assess the discriminatory power of different genes in deriving the four disease classes through classification. The classifier showed good prediction accuracy (79.6%, OOB error rate 20.37%, 1000 trees). Figure 4 shows the MDS plot for this classification experiment, illustrating the relative clustering of samples in four skin phenotypes. As before, non-inflamed tissues (NN and PN) clustered away from the inflamed tissues (PP). Additionally, the relative segregation of the two PP subgroups was also apparent.

**Figure 5:**
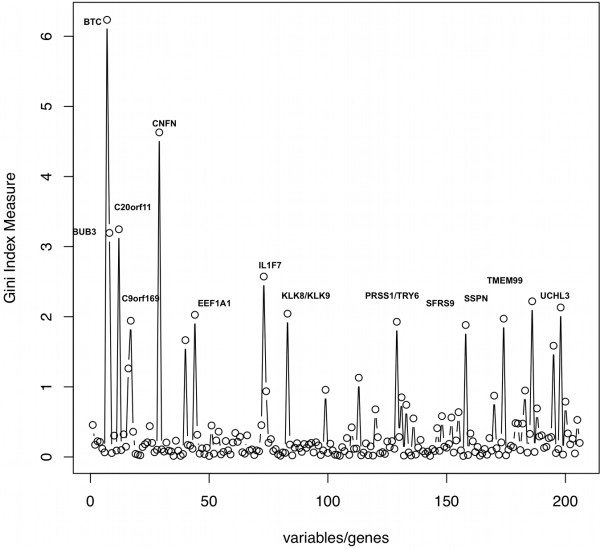
**Genes identified as most informative through RF classification of skin tissues in four molecular groups (NN, PN, PP01 and PP02).** Gini Index (GI) was used as variable importance measure for estimating the discriminative power of relevant features (genes) and, consequently, their relevance to disease state. The five most important genes in determining disease classes were *BTC*, *CNFN*, *C20orf11*, *BUB3* and *IL1F7.* Genes with related annotation are listed in Table 1.

The contribution of particular genes in differentiating the corresponding disease phenotypes was also assessed through Gini Index as variable importance measure and Figure 5 illustrates a measure of the discriminative power of genes in classification. Functional information of the five top-scoring genes is listed in Table 1, in terms of chromosomal location, GO class and pathway participation. This set of most informative genes in Figure 5 and Table 1 was also confirmed by calculating the empirical p-value by permutating the tissue labels [33] (see Methods).

**Table 1:** Functional annotation for most informative genes

***BTC***	**Description:** betacellulin
	**Chromosomal region:** chr4q13-q21
	**GO-BP:** cell proliferation, positive regulation of cell proliferation
	**GO-MF:** epidermal growth factor receptor binding, growth factor activity, growth factor activity
	**GO-CC:** extracellular region, soluble fraction, plasma membrane, integral to membrane
	**Pathway:** ErbB signalling, ERK signaling
***CNFN***	**Description:** cornifelin
	**Chromosomal region:** chr19q13.2
	**GO-BP:** keratinization
	**GO-MF:** NA
	**GO-CC:** cornified envelope, cytoplasm
	**Pathway:** NA
***C20orf11***	**Description:** Protein C20orf11 (two hybrid-associated protein 1 with RanBPM) (Twa1)
	**Chromosomal region:** chr20q13.33
	**GO-BP:** NA
	**GO-MF:** protein binding
	**GO-CC:** nucleus
	**Pathway:** NA
***BUB3***	**Description:** budding uninhibited by benzimidazoles 3 homolog (yeast)
	**Chromosomal region:** chr10q26
	**GO-BP:** mitosis, cell proliferation, anaphase-promoting complex-dependent proteasomal ubiquitin-dependent protein catabolic process, negative regulation of ubiquitin-protein ligase activity during mitotic cell cycle
	**GO-MF:** protein binding
	**GO-CC:** kinetochore, nucleus, cytosol
	**Pathway:** Cell cycle role of APC in cell cycle regulation
***IL1F7***	**Description:** interleukin 1 family, member 7 (zeta)
	**Chromosomal region:** chr2q12-q14.1
	**GO-BP:** immune response
	**GO-MF:** cytokine activity, interleukin-1 receptor binding, interleukin-1 receptor antagonist activity
	**GO-CC:** extracellular region
	**Pathway:** Systemic lupus erythematosus signaling, role of cytokines in mediating communication between immune cells, graft-versus-host disease signaling, p38 MAPK signaling, atherosclerosis signaling

To extract the differences in gene expression between the two psoriasis sub-groups, we generated a co-expression network of the core genes for each of these groups (Additional file 4: Figure S2, see also Methods). This resulted in two networks for PP01 and PP02 with different topological properties. The PP01 network consisted of 122 nodes with 142 edges, whilst PP02 had 173 nodes with 472 edges. After clustering with MCL [34], 36 clusters were identified for each patient group, PP01 and PP02, and functional enrichment analysis of the largest clusters has indicated different biochemical pathways linked to each of these groups. The PP01 network clusters were enriched in signaling pathways, such as Wnt, Notch, TGF-beta, ErbB signaling pathways, whereas clusters in PP02 network were more involved in metabolic pathways (Tables 2 and 3). This indicated that the two lesional psoriatic sub-groups possess different functional properties, suggesting different underlying biological processes.

**Table 2:** Pathway enrichment in the PP01 psoriatic group

**Pathway name**	**p-value**	**q-value**
**Cluster1**		
NOTCH1 Intracellular Domain Regulates Transcription	0.0008	0.0227
Signaling by NOTCH1	0.0017	0.0254
Signaling by NOTCH	0.0028	0.0275
NOTCH1 Intracellular Domain Regulates Transcription	0.0007	0.0227
**Cluster2**		
Urea cycle	0.0066	0.0187
Synthesis of very long-chain fatty acyl-CoAs	0.0104	0.0187
Fatty Acyl-CoA Biosynthesis	0.0133	0.0187
Triglyceride Biosynthesis	0.0271	0.0286
**Cluster3**		
PI3K events in ERBB4 signaling	0.0003	0.0054
PI3K events in ERBB2 signaling	0.0004	0.0054
Signaling by ERBB4	0.0019	0.0142
Signaling by ERBB2	0.0024	0.0142
AKT phosphorylates targets in the nucleus	0.0059	0.0289
Signaling by TGF beta	0.0106	0.0400
SHC1 events in ERBB4 signaling	0.0139	0.0400
GRB2 events in ERBB2 signaling	0.0152	0.0400
Signaling by BMP	0.0159	0.0400
SHC1 events in ERBB2 signaling	0.0165	0.0400
PIP3 activates AKT signaling	0.0205	0.0434
Immune System	0.0215	0.0434
PI3K/AKT activation	0.0263	0.0447
Nuclear signaling by ERBB4	0.0273	0.0447
GAB1 signalosome	0.0283	0.0447
Interleukin-1 signaling	0.0296	0.0447

**Table 3:** Pathway enrichment in the PP02 psoriatic group

**Pathway name**	**p-value**	**q-value**
**Cluster2**		
Transport of Glycerol from Adipocytes to the Liver by Aquaporins	0.0018	0.0158
Transport by Aquaporins	0.0102	0.0419
Signaling by TGF beta	0.0149	0.0419
Signaling by BMP	0.0223	0.0470
**Cluster3**		
Respiratory electron transport	0.0012	0.0009
Respiratory electron transport, ATP synthesis by chemiosmotic coupling, and heat production by uncoupling proteins.	0.0018	0.0009
The citric acid (TCA) cycle and respiratory electron transport	0.0036	0.0013

### Identification of key genes associated with disease sub-classes and comparison with other studies

**Figure 6:**
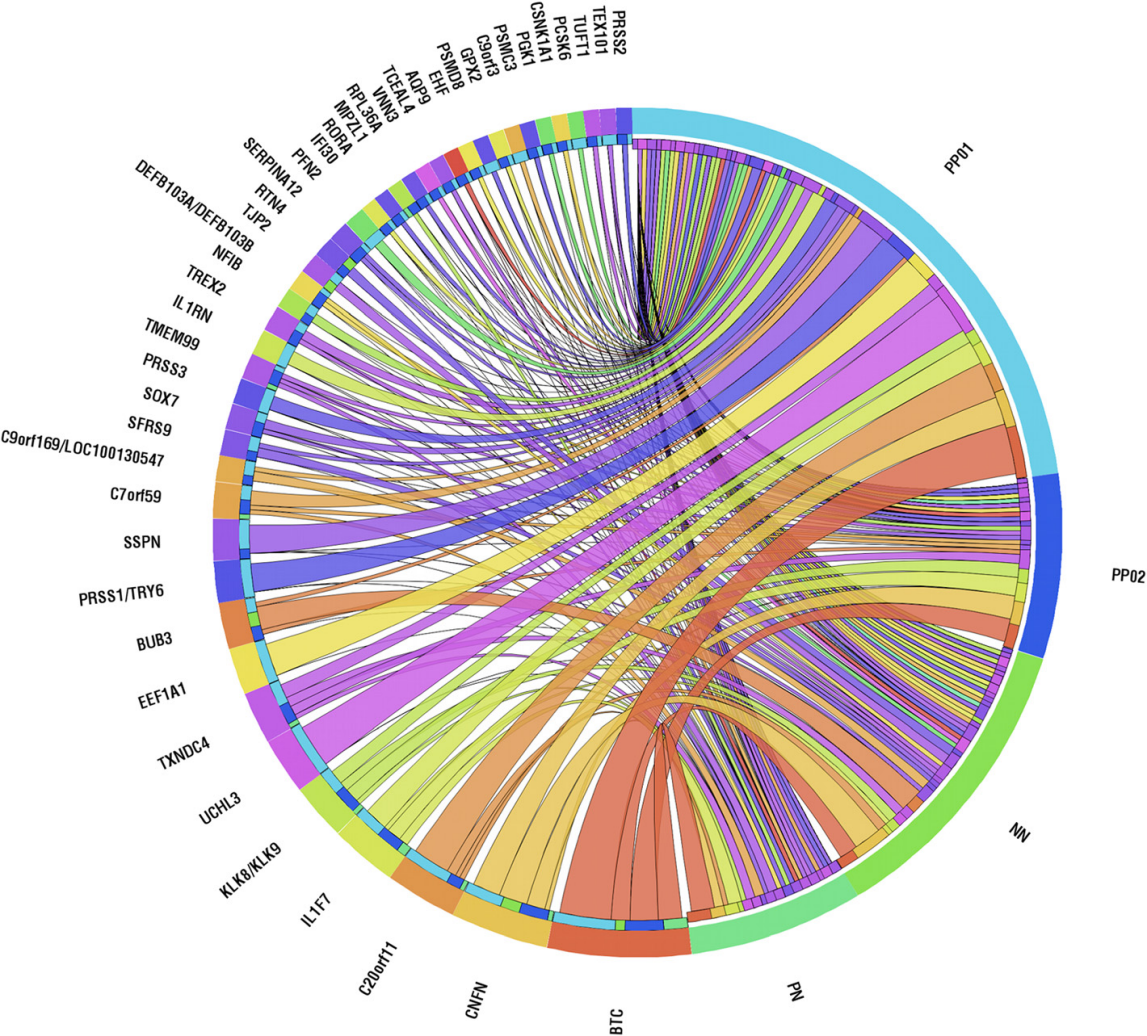
**Graphical representation to illustrate the relation between 43 highly discriminative genes and disease sub-groups.** Contributions shown according to Gini Index, calculated from random forest classification. The four skin-types (PP01: light blue, NN: green, PP02: blue, PN: light green) followed by relevant genes are arranged clockwise. Skin groups and genes are ordered according to shared pairing links.

Variable importance analysis was used to derive highly discriminative genes in classification of disease sub-types. As described previously (see Methods), the Gini Index (GI), calculated from RF classification (108 samples, 206 genes, 4 classes, see Figure 1, *Step 3*), was used to rank each of the 206 genes in terms of their discriminatory effect in assigning samples in each of the four tissue groups (NN, PN, PP01, PP02). The 43 genes with highest GI were schematically represented in a circular layout (Figure 6) to show their effect in each of the four classes. For example, *BTC*, part of the ErbB and ERK Signaling pathways, has GI equal to 0.11 for samples classified in the PP01 class and a significantly lower GI when classifying samples in the other tissue groups (GI = 0.041 for NN, GI = 0.056 for PN and GI = 0.055 for PP02). Similarly, the importance of the other 43 genes in classifying samples in the four phenotype classes was determined and is illustrated (Figure 6).

**Figure 7:**
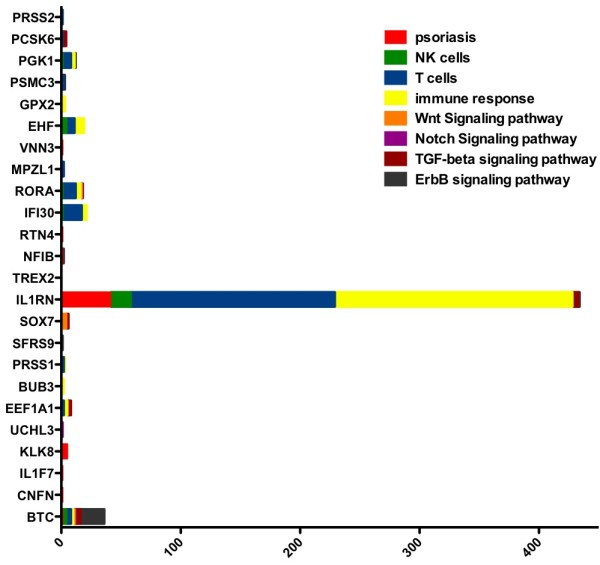
**Text mining results for validation process according to the literature.** Co-occurrence of gene names with disease-related terms, such as”psoriasis”, “NK cells”, “T cells”, “immune response”, “Wnt signaling pathway”, “Notch signalling pathway”, “TGF – beta signaling pathway” and “ErbB signaling pathway” was searched in Pubmed abstracts through PubMatrix.

In the NN group, *CNFN* and *BUB3* were more frequently selected to define a split in the classification trees of the forest, whereas *BTC*, *IL1F7* and *TMEM99* (GI > 0.02) were important in classification of PN samples. Within the PP group, *BTC*, *C20orf11*, *EEF1A1*, *CNFN*, *IL1F7*, *PRSS1*/*TRY6*, *SSPN* and *UCHL3* showed high discriminative value for identifying the PP01 sub-group, whereas *BTC*, *CNFN*, *IL1F7*, *KLK8*/*KLK9* and *TXNDC4* exhibited high importance for the PP02 sub-group (Additional file 5: Figure S3). To further support linking these genes to psoriasis-related biological mechanisms, the PubMatrix tool was used to look up the discriminatory genes in the context of eight terms, including”psoriasis”, “NK cells”, “T cells”, “immune response”, “Wnt signaling pathway”, “Notch signaling pathway”, “TGF – beta signaling pathway” and “ErbB signaling pathway” [35]. Out of 43 genes, 24 genes were found to occur together with these terms in biomedical literature, as seen in Figure 7 (see also Additional file 6: Table S2). Interestingly, BTC, which exhibited a high discriminative value when characterising PP01 sub-type, was found to be related with the ErbB signaling pathway. The latter was a highly enriched pathway in this sub-group and indicates a potential therapeutic target. IL1RN also had a high GI for samples classified as PP01 and was previously found to be highly related with T cell activation and immune response.

The pipeline outlined above was replicated with two other psoriatic datasets from [18] (Gudjonsson dataset) and [36] (Yao dataset). Skin samples were grouped into sub-types according to their gene expression patterns as for the GAIN dataset, using similarities derived from the proximity matrix through random forest (an MDS plot for Gudjonsson and Yao data is shown in Additional file 7: Figure S4). The circular representation of the most important genes was also followed here and the 19 most informative genes from Gudjonsson and 27 from Yao datasets are shown (Additional file 8: Figure S5 and Additional file 9: Figure S6). By comparing across the three datasets and the relevant gene signatures, the importance of specific genes was noted. *BTC*, *CNFN*, *IL1F7* were important discriminant genes in the GAIN data, while *SNRPC* and *SMURF2* played a greater role in the Yao and Gudjonsson datasets. Generating a consistent outcome of gene signatures across all datasets is challenging, as patient cohorts may vary significantly. Although the Yao data seem difficult to reproduce, considerable similarity exists between the other two datasets. Specifically, one of the disease subgroups in these dataset points to pathways related to NOTCH signaling, ErbB and TGF beta suggesting that this group may be more amenable to related therapeutic options (see Tables 2, 3, Additional file 10: Table S3 and Additional file 11: Table S4).

We note that evaluation of psoriasis transcriptomes has been assessed elsewhere [20] and the observed low reproducibility across various studies was attributed to wide variability in clinical protocols, platforms and sample handling among different datasets. It is envisaged that the application of the present and similar strategies for predictive modelling and stratification of expression patterns, as well as the availability of larger patient studies will bridge the disparities between various studies and yield a sharper picture of gene contributions to this complex disorder.

## Conclusions

Large-scale genome characterisations, through the analysis of gene sequence and expression data, are gaining increasing interest and have the potential to greatly improve our understanding of the physiological and molecular mechanisms underlying disease pathogenesis and progression. Various models of data stratification and identification of patient groups through various data mining protocols are used to support a decision making process in biomedicine. Data mining procedures based on algorithms such as support vector machines (SVM), neural networks, decision tree algorithms and mathematical programming approaches have been used to select sets of genes for diagnostic purposes and to identify molecular roles which are - as yet- unknown [37]. Here we have illustrated the use of random forest to partition psoriatic tissues in appropriate disease groups and generate estimates of relevant gene predictors.

Psoriasis is a common, complex immuno-genetic inflammatory disease of primarily the skin. The underlying genetics of the disease are complex with numerous implicated susceptibility genes, where replication of single loci has been confirmed for only a handful of these genes. Patients suffering from psoriasis can exhibit a host of different clinical phenotypes and response to therapy is varied and unpredictable, even within a similar clinical phenotype, suggesting underlying transcriptional differences between and within the clinical groups. The ability to investigate the underlying immuno-genomic components of these clinical sub-phenotypes has not been a possibility, until now. Identification of different transcriptional signatures and their associated molecular pathways contribute toward defining a set of biomarkers, which could serve as diagnostic and therapeutic responder tools. We have outlined a computational strategy to identify molecular sub-types and corresponding putative biomarkers that may be crucial in the understanding and prediction of disease pathogenesis. Of the 206 common differentially expressed genes identified between normal, psoriatic lesional and psoriatic non-lesional groups, 130 genes (63.1%) were up-regulated and 76 genes (36.9%) were down-regulated. Dysregulated genes discovered in this study were involved in epidermal cell modulation, cell cycling and immune responses.

Microarray analysis of gene expression has been widely used to differentiate lesional and non-lesional skin of psoriatic patients [38,39]. Recently, large-scale analysis using whole genome array platforms on numerous patients per sample group have been undertaken with the aim of identifying gene expression profiles associated with a specific psoriatic phenotype [5,6,10,40]. In this work, we present a method for identifying sub-phenotypes of lesional skin from psoriasis patients based on patterns of gene expression that characterise each group and differ significantly from normal human skin. This approach is based on a decision tree analysis of gene expression data, the extraction of associations among gene expression patterns and the identification of functional annotations and molecular signatures.

The random forest decision tree model was applied to lesional skin group to derive patient sub-groups (PP01 and PP02), which are characterised by specific differentially expressed genes. The PP01 group was defined by the up-regulation of HLA-E, which is the inhibitory ligand for innate NK cells. HLA-E takes part in processing and presenting antigen to innate immune cells. The PP02 group had more up-regulated genes related to the cells of the adaptive immune system such as *CTLA-4* (associated with modulation of T helper responses), *IFI30* (involved in MHC Class II antigen processing), *IL4IL* (immunomodulatory enzyme produced by dendritic cells), *PTPN2* (associated with autoimmune disorders such as type 1 diabetes mellitus and Crohn’s disease) and most interestingly *SERPINB8*, which has been identified through Genome-Wide Association Studies (GWAS) as a new psoriasis susceptibility locus in the Chinese population [41].

With regards to mechanistic details on the pathways that operate in psoriatic sub-groups, the ErbB signaling pathway has been identified for subgroup PP01 (Table 2). This pathway consists of a family of four related receptor tyrosine kinases (ErbB1-4) which, when activated trigger many different signal transduction pathways leading to increased proliferation, survival, motility, and invasiveness [42]. All of these responses are important aspects of wound healing and psoriasis has many elements in common with wound healing. The main clinical feature of psoriasis relates to the thickened epidermis as a result of what may initially have been an epidermal barrier insult. An attempt to restore epidermal integrity is reflected in the activation of the ErbB signaling pathway. However in psoriasis it is possible that this pathway, along with other signaling pathways is dysregulated [43].

Other signaling pathways seem to be in effect in psoriasis sub-group PP02 (Table 3), for example signaling by BMP. Bone morphogenetic proteins (BMP) are members of the transforming growth factor-beta (TGF beta) superfamily and regulate a large variety of biological responses in different cells and tissues. It has been reported that BMPs are implicated in a variety of pathobiologic processes in skin, including wound healing, psoriasis, and carcinogenesis [44].

In our analysis, when several patient clinical variables were compared across the two classes (PP01 and PP02), we found age to be significantly altered in these subgroups, indicating that this is an important factor in disease manifestations. It is worth noting that although the differences in PP01 and PP02 groups are quite marked on a transcriptional level, yet they are clinically difficult to distinguish. This observation may help explain why some patients have a different disease course to others and why some respond better to therapy than others within a given clinical phenotype. The ability to generate molecular sub-types provides putative biomarkers, which with further refinement and replication, could prove to be useful in predicting disease severity, progression and response to therapy in an individualised manner.

Random forest has become a popular tool for analysing high-throughput genomic data. Due to the large number of variables associated with characterisation of clinical samples through gene expression measurements, reduction of dimensionality through feature selection or prioritisation is critical in disease property prediction. Here, we use random forest for (i) disease classification through gene expression patterns and analysis of variable importance to generate potential disease biomarkers, and (ii) clustering of gene expression measurements to derive disease subgroups. Despite some limitation in reproducibility across different psoriasis datasets, we believe that through our study there is an emerging picture of important gene predictors in psoriasis, as well as differentiation of disease in patient subgroups. Future work based on richer datasets that profile larger patient cohorts, with stringent clinical phenotyping, will have the potential to draw clearer conclusions about this complex autoimmune skin disease.

In this study, we generated biologically meaningful phenotypic classes using a ‘core’ of the highest differentially expressed genes and then further addressing the molecular variations among the groups responsible for lesional psoriasis. This might uncover subtle differences in disease pathogenesis allowing the emergence of new treatments for psoriatic individuals and further facilitate the development of personalized treatments for the disease. To the best of our knowledge, this is the first analysis identifying substantial phenotypic groups in psoriasis, based on patient gene expression profiles and using a classification pipeline. Further analysis and discovery of patterns and associations of transcripts of different cell-types (such as T-cells, dendritic cells, keratinocytes) must be done to shed light on the contribution that different cell types make towards the pathogenesis of psoriasis. We would then gain a better insight into this unique skin disease and hopefully, resolve some of the outstanding issues related to its pathogenesis and treatment.

## Methods

### Data sources

Microarray data on psoriatic gene expression were obtained from the Genetic Association Information Network (GAIN) Database [10,45], available through the NCBI database of Genotypes and Phenotypes (dbGaP). These experiments describe tissue samples from 71 individuals, of which 34 were healthy controls (NN) and 37 patients affected by chronic plaque psoriasis. Paired samples from lesional (PP) and non-lesional (PN) tissues were extracted and gene expression was measured by microarray experiments on the Affymetrix HU133 Plus 2.0 platform. Raw data were normalized using quantile normalization and expression estimates were computed using the Robust Multichip Average (RMA) method [46].

Analyses performed on the above gene expression dataset were validated through comparison with gene expression datasets GSE14905 and GSE13355 from the ArrayExpress database [47]. The first study consisted of 21 biopsies from healthy donors and 26 paired non-lesional and lesional plaque type psoriatic patients [36] and the second dataset comprised 64 normal samples and 58 psoriatic tissues [10,18]. Both studies were conducted on hgu133plus2 Affymetrix chips.

### Differential expression analysis

In order to define a ‘core’ dataset of differentially expressed genes in the psoriatic phenotypes examined, pairwise comparisons between 34 normal (NN) and 37 lesional (PP) and non-lesional (PN) gene expression vectors were performed. The differential expression between pairs of samples (PP vs. NN, PN vs. NN, PP vs. PN) was assessed using GenePattern [48]. Significance scores were assigned to each probe (p-value < 0.05), multiple hypothesis testing was applied with FDR < 0.05 to reduce the false positives and the top ranked 5000 probes were extracted for each pair of samples. Of those, the set with the most common expression alteration among the three pairwise comparisons was selected. Probes that mapped to the same gene were averaged and the average intensity across all corresponding genes was used. A core set of 228 probes common to all three pairwise comparisons was established. Of these, a total number of 206 unique known genes were derived yielding 130 up-regulated and 76 down-regulated genes (Additional file 1: Table S1).

Hierarchical clustering and principal component analysis (PCA) were implemented to identify distinct patterns of gene expression within the ‘core‘206 differentially expressed genes. The PCA procedure was implemented as part of the PCA package in R (http://www.r-project.org). Unsupervised hierarchical clustering heat-maps were generated in R based on Euclidean distance. Z-scores were calculated from the level of normalized expressions of 206 genes according to the mean and standard deviation of a reference set (control samples, NN).

### Decision tree classification model

An ensemble of decision trees model was built according to the random forest (RF) classifier using a deterministic algorithm (Classification and Regression Tree Algorithm, CART) [22]. Given a gene expression matrix, a RF classifier was constructed to classify tissue samples into relevant disease classes (NN, PN, PP) based on gene expression measurements (variables). Details on the classification strategy are given in Supplementary Methods and a small example of the classification process is shown in Supplementary Information (Additional file 12: Figure S7). Variable importance measures were implemented through mean decrease in accuracy and the Gini Index (GI) [24], to find the genes that best discriminate between the different disease phenotypes. Both measures were tested and have been found to correlate well (Additional file 13: Figure S8). The Gini Index was adopted to express the relative effects of gene predictors in determining the relevant disease classes. To estimate the empirical p-value for GI, 1000 permutations of the tissue samples were implemented and the importance values were recalculated for the permuted data set. The maximum Gini Index over all the genes in every permutation was recorded and thereby an empirical distribution of the maximum importance was estimated, as in similar analyses [49,50].

### Clusters of disease sample sub-groups through decision tree classification

A procedure to generate clusters of disease samples from gene expression measurements through the use of RF is described here. The random forest proximity measure, defined through the number of times each tree detects these samples in the same terminal node, is used as a means to express the similarity between samples from gene expression observations (Additional file 2: Supplementary Methods). Psoriatic microarray data were used to generate molecular sub-types. Synthetic data are generated by randomly sampling the empirical marginal distributions of variables. RF classification is applied to distinguish the 37 psoriatic samples from the synthetic data and the dissimilarity matrix is used to indicate distances between psoriatic samples, as previously [29]. Through multi-dimensional scaling, samples are represented as points before clustering through CLARA [51]. This procedure was implemented in R. Statistical significance of disease clusters with respect to clinical variables was done through Wilcoxon signed-rank test and the clinical variables tested were Psoriasis Area and Severity Index (PASI), Body Mass index (BMI), Age of Onset, Age and Body Surface Area (BSA).

### Network analysis and functional enrichment

Pairwise Pearson‘s correlation coefficient is estimated for the 206 differentially expressed genes that were common in all tissues. A similarity matrix was calculated for each skin sub-type and a co-expression network was visualised using the Cytoscape software. Markov Cluster Algorithm (MCL) was used to generate the interacting groups (clusters) via genes sharing higher-order connectivity in their local neighborhoods [34]. To assess statistically significant enriched pathways involved in the four different skin groups, p-values were calculated using the hypergeometric statistical test and False Discovery Rate (FDR < 0.05) was used to correct for multiple comparisons. The default background distribution is considered to be the whole genome. Pathway enrichment analysis was performed using the ReactomePA package in Bioconductor [52,53].

### Collaborative Association Study of Psoriasis

Support for genotyping of samples was provided through the Genetic Association Information Network (GAIN). The dataset used for the analyses described in this manuscript were obtained from the database of Genotypes and Phenotypes (dbGaP) found at http://www.ncbi.nlm.nih.gov/gap through dbGaP accession number phs000019.v1.p1. For samples and associated phenotype data, we kindly acknowledge the Collaborative Association Study of Psoriasis and Profs. J.T. Elder, J. Ding, W. Swindel, G. Abecasis, P. Stuart and R. Nair.

## Competing interests

The authors declare that they have no competing interests.

## Authors’ contribution

CA, NV and ST conceived and designed the experiments, CA performed the experiments, CA, NV, GP, AW, JEG, CAO, FON, ST analysed data, CA, GP, CAO, FON, ST wrote the paper. All authors read and approved the manuscript.

## Supplementary Material

Additional File 1 The ‘core’ set of genes defined through differential expression analysis: positively (130) and negatively (76) differentially expressed genes in psoriatic samples of the GAIN dataset. Click here for file

Additional File 2 Supplementary methods. Click here for file

Additional File 3 A multidimensional scaling (MDS) plot showing the distinction of psoriatic cases into two groups, PP01 (red) and PP02 (black), as obtained after RF clustering and classification. Click here for file

Additional File 4 **Markov Cluster Algorithm (MCL) applied on the psoriatic sub-group tissue sample networks to extract clusters of gene expression.** Both networks consisted of 36 clusters and the largest clusters (number of nodes > 8) for both networks are shown and denoted by colour. Pathway enrichment for these clusters is shown in tables 3 and 4 for PP01 and PP02 networks respectively. Click here for file

Additional File 5 **Genes identified as most informative after classification of skin disease phenotypes.** Gini Index (GI) was used as variable importance measure and was estimated for each gene per group from random forest classification, so as to prioritise genes in terms of their ability to discriminate distinct molecular patterns. After training of the random forest classifier, GI is derived for each gene across all trees and the ranking of genes with GI > = 0.02 is shown here for each skin group. Click here for file

Additional File 6 Results of text mining. Click here for file

Additional File 7 **A multidimensional scaling plot of psoriasis datasets from Gudjonnson et al. 2010 [18] (A) and Yao et al. 2008 [36] (B) to illustrate grouping of samples according to random forest clustering.** Two distinct psoriatic groups are identified in involved tissue (PP01 green and PP02 purple), while NN and PN samples largely co-localise. Overall, clustering is comparable to GAIN data that is shown in figure 4. Click here for file

Additional File 8 **Graphical representation to illustrate the relationship between 19 highly discriminative genes and disease sub-groups according to Gini Index calculated from decision trees forest in the Gudjonnson dataset.** The green band represents the first psoriatic group (PP01), light blue corresponds to the second psoriatic sub-group (PP02), yellow corresponds to healthy individuals (NN) and light green presents the non-lesional cases (PN) and are arranged clockwise followed by purple to orange rectangular bands that represent relevant genes. Genes and skin groups are ordered according to shared pairing links, as described previously. Click here for file

Additional File 9 **Graphical representation to illustrate the relationship between 27 highly discriminative genes and disease sub-groups according to Gini Index calculated from RF for the Yao dataset. **Light blue to green rectangular bands represent the four skin-types (PP01: light blue, PP02: blue, NN: light- green, PN: green) and are followed by purple to orange rectangular bands representing relevant genes (arranged clockwise). Genes and skin groups are ordered according shared pairing links. An overview of patterns of informative genes for prediction of each disease class can be visualised. Click here for file

Additional File 10 Pathway enrichment in the Gudjonsson dataset. Click here for file

Additional File 11 Pathway enrichment in the Yao dataset. Click here for file

Additional File 12 **Example of a decision tree for classification of tissue samples in appropriate disease classes.** Heatmap illustrates expression values for 25 genes across 108 tissue samples and represents part of the heatmap shown in figure 2. A decision tree is a tree-like structure to relate gene expression measurements to sample phenotype class, with a view to deriving a predictive model. Nodes (rectangles) in the tree represent a test on gene expressions to derive a decision on a sample’s class, edges (arrows) indicate the expression level of the variable that can best distinguish the samples and leaves (or terminal nodes - circles) represent class predictions. The path from root to each terminal node equates to a list of conditions in the form of gene expression rules that can relate tissue samples to disease phenotype class. Click here for file

Additional File 13 Correlation between the two variable importance measures of gene selection, Gini Index and mean decrease in accuracy. Click here for file
