# Supplementary material for: Transcriptome classification reveals molecular subtypes in psoriasis
Source: BMC Genomics. 2012 Sep 12;13:472. doi: 10.1186/1471-2164-13-472 (PMC3481433; doi:10.1186/1471-2164-13-472)
Supplement: Additional File 1 — The ‘core’ set of genes defined through differential expression analysis: positively (130) and negatively (76) differentially expressed genes in psoriatic samples of the GAIN dataset. [file 1471-2164-13-472-S1.doc]

**Table S1:** The ‘core’ set of genes defined through differential expression analysis: positively (130) and negatively (76) differentially expressed genes in psoriatic samples of the GAIN dataset.

| **Down** (76) | AQP9,BBS1,BBX,BTC,C11orf49,C11orf67,C1orf96,C2orf55,C6orf26/MSH5,C7orf59,CAT,CD81,CGNL1,CMTM4,CORO2B,CRTC3,DCD,DIXDC1,DYNLRB1,EEF1A1,EIF4E3,FAM174B,FLG2,FN1,GREM2,IL1F7,IRS2,KCMF1,KIAA1704,LAPTM4A,LOC202451,LOC400960,LRRC17,LYPD6B,LZTS2,MEGF9,MTMR3,MYH10,NCOR2,NFIB,PALM,PARD3,PCYOX1,PDGFC,PELI3,PFN2,PHF2,PIGL,PPP1R3B,PTPN21,RFESDRORA,RPL36A,RPL7,RRAGD,RTN4,SCARB2,SERPINA12,SLC25A36,SLC46A2,SMURF2,SPIN1,SSPN,SVIP,SYN2,TBC1D24,TCEAL4,TCF7L1,TMEM99,TRIM45, TUBGCP4,TUFT1,YIPF6, ZFP28,ZKSCAN1,ZNF12 |
| --- | --- |
| **Up** (130) | ARG1,ARPC5L,ATP11B,BUB3,C20orf11,C22orf28,C9orf169/LOC100130547,C9orf3,CALML5,CBR1,CCDC19,CCT7,CDC25A,CIP29,CNFN,COMTD1,COQ2,CRCT1,CRIPT,CSNK1A1,CTLA4,D4S234E,DEFB103A/DEFB103B,DUSP23,EHF,ELOVL7,EPN3,EREG, EXOSC1,FAM62C,FBXO6,FKBPL,FLJ21511,FUT1,FUT5 /NDUFA11,GBA /GBAP,GBP2,GNAI3,GPX2,H2AFJ,HAL,HLA-E,HSD17B10,HSPC152,HTATIP2, ICHTHYIN,IFI30,IL1RN,IL4I1,KARS,KLHL18,KLK7,KLK8,KLK8/KLK9,KPTN,KRTDAP,LACTB,LOC100134440/PGBD5,LRP8,LSMD1,LTB4R2,LYNX1,MPZL1,MRPL23,MRPL46,MRPL54,MRPS18C,MYEOV2,NDUFA13,NDUFA7,NDUFB2,NDUFB8,NFASC,PCSK6,PGK1,PLCXD1,PON2,PPARD,PRSS1,PRSS1/TRY6,PRSS2,PRSS3,PSMA1,PSMC3,PSMC5,PSMD13,PSMD8,PSMD9,PTGER3,PTPN2,RBX1,RNASE7,RNASEH2A,ROBLD3,RPL22L1,RPS6KB2,SDR9C7,SERPINB8,SF3B14,SFRS9,SHFM1,SMPD3,SNAP29,SNRPA1,SNRPC,SNRPD1,SNRPD3,SNRPG,SOX7,SQLE,SSNA1,TC2N,TCEB1,TEX101,TJP2,TMEM54,TMEM86A,TRAPPC5,TREX2,TSEN15,TSFM,TTC1,TXNDC4,UBE2D3, UBL5,UCHL3,UGCG,VNN3,WDR4,WDR53 |
