# Supplementary material for: Transcriptome classification reveals molecular subtypes in psoriasis
Source: BMC Genomics. 2012 Sep 12;13:472. doi: 10.1186/1471-2164-13-472 (PMC3481433; doi:10.1186/1471-2164-13-472)
Supplement: Additional File 2 — Supplementary methods. [file 1471-2164-13-472-S2.doc]

**SUPPLEMENTARY METHODS**

Random forest classification model

An ensemble of decision trees model was built according to the random forest (RF) classifier using a deterministic algorithm (Classification and Regression Tree Algorithm, CART) . Given an expression matrix as training set X with G variables/genes and S samples/patients that belong to three classes (NN, PN, PP), a decision tree was constructed. For each variable g and for each expression value v, we estimate the best criterion to maximally partition the space and best distinguish S samples. Using the divide and conquer strategy to recursively dissect the data, the best condition is selected to partition the space into two parts, Xr and Xl according to the threshold g<v. The splitting procedure is repeated in order to obtain pure regions, where no further splitting is possible. A small example of the classification process is shown in Figure S5.

Random forest (RF) takes advantage of two key machine-learning techniques: bagging (bootstrap aggregation) and random feature selection. In bagging, each tree is trained on a random subset of samples drawn with replacement, resulting in about 2/3 of samples being part of the training set (in-bag data) and the remaining 1/3 of samples constitute the testing set (out-of-bag data, OOB), where prediction performance is estimated. As the tree grows at each step, a subset of variables is selected at random without replacement so as to achieve the best split at each node. To assess the prediction performance of the RF algorithm, OOB samples are used for the testing procedure, resulting in a type of k-fold cross-validation method. Overall, using different subsets of training data and features, the classifier can effectively propose a predictive model that links disease samples to appropriate phenotypes through relevant gene expression patterns. RF analysis was carried out through the R software – RF package, version 4.5-3 3 .

Variable Importance Measures

Variable importance measures in classification experiments can serve as means to quantify the effect of each variable (i.e. gene) in discriminating among the relevant class outcomes (phenotypes). RF returns two measures of variable importance, mean decrease in accuracy and the gini index . Mean decrease in accuracy is motivated from statistical permutation tests and is based on the decrease of prediction accuracy when values of a variable in a node of a tree are permuted randomly . The gini index measure (GI) is derived from training of the RF classifier and is used as the splitting criterion in the construction of the trees . Measures were standardized and normalized over all trees.

Both measures were tested and have been found to correlate well (Figure S6). Gini index (GI) was adopted to measure the value of each gene expression profile for class differentiation. At each node within the trees of the random forest, the optimal split is sought using the Gini index – a computationally efficient approximation to the entropy – measuring how well a potential split is separating the samples of the two classes in this particular node . We therefore defined a scoring system, ranked the candidate genes and identified biological indicators (biomarkers) that are discriminatory between groups of samples.

Clusters of disease sample sub-groups through decision tree classification

A procedure to generate clusters of disease samples from gene expression measurements through the use of RF is described here. Central in this respect is the random forest proximity measure, used as a means to express the similarity between samples from gene expression observations. Through the construction of RF predictor, counting the number of times each tree detects these samples in the same terminal node generates a proximity measure between samples. Dividing the proximity measure by the number of trees normalizes similarities between samples and a relevant matrix is formed () with values [0,1]. Dissimilarity is defined as .

Psoriatic microarray data were used to generate molecular sub-types. Synthetic data are generated by randomly sampling the empirical marginal distributions of variables. RF classification is applied to distinguish the 37 psoriatic samples from the synthetic data and the dissimilarity matrix is used to indicate distances between psoriatic samples, as previously . Through multi-dimensional scaling, samples are represented as points before clustering through CLARA . This procedure was implemented in R. Similarity of the defined molecular sub-types were measured with Rand Index metric. Statistical significance of disease clusters with respect to clinical variables was done through t-test multigroup comparison tests.

**REFERENCES**

1. Becker KG, Hosack DA, Dennis G, Jr., Lempicki RA, Bright TJ, et al. (2003) PubMatrix: a tool for multiplex literature mining. BMC Bioinformatics 4: 61.

2. Breiman L, Friedman JH., Olshen RA., CJ. S (1984) Classification and Regression Trees. New York: Chapman and Hall.

3. Liaw A, Wiener M (2002) Classification and Regression by RandomForest. . R News 3: 18-22.

4. Diaz-Uriarte R, Alvarez de Andres S (2006) Gene selection and classification of microarray data using random forest. BMC Bioinformatics 7: 3.

5. Strobl C, Boulesteix AL, Kneib T, Augustin T, Zeileis A (2008) Conditional variable importance for random forests. BMC Bioinformatics 9: 307.

6. Menze BH, Kelm BM, Masuch R, Himmelreich U, Bachert P, et al. (2009) A comparison of random forest and its Gini importance with standard chemometric methods for the feature selection and classification of spectral data. BMC Bioinformatics 10: 213.

7. Johannes L, Grabmeier , Lambe LA (2007) Decision trees for binary classification variables grow equally with the Gini impurity measure and Pearson's chi-square test. International Journal of Business Intelligence and Data Mining v.2 n.2: p.213-226.

8. Shi T, Seligson D, Belldegrun AS, Palotie A, Horvath S (2005) Tumor classification by tissue microarray profiling: random forest clustering applied to renal cell carcinoma. Mod Pathol 18: 547-557.

9. Kaufman L, Rousseeuw PJ (1990) Finding groups in data: an introduction to cluster analysis. New York: Wiley.
