# Supplementary figures and images for: Transcriptome classification reveals molecular subtypes in psoriasis
Source: BMC Genomics. 2012 Sep 12;13:472. doi: 10.1186/1471-2164-13-472 (PMC3481433; doi:10.1186/1471-2164-13-472)

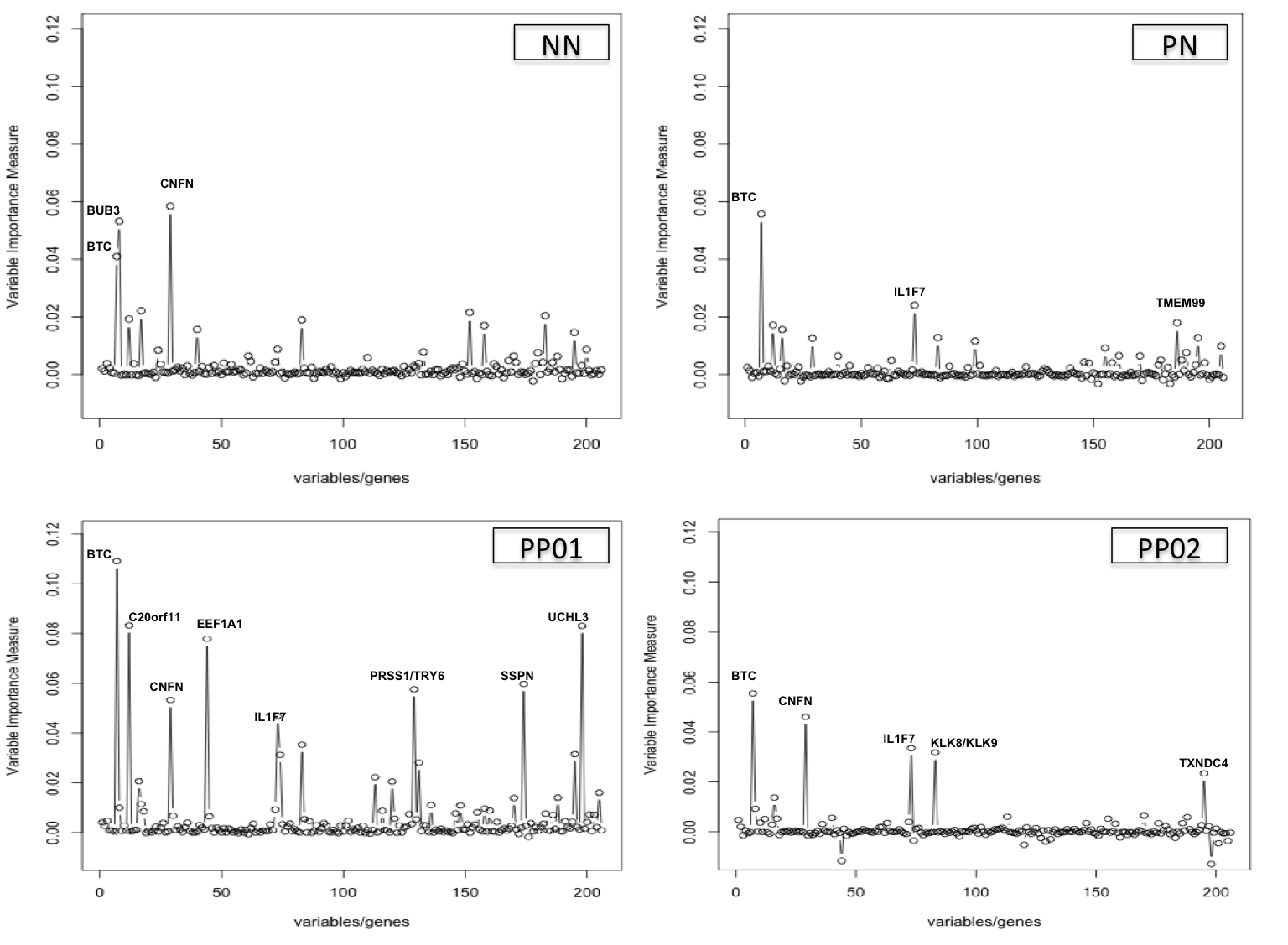

Supplement: Additional File 3 — A multidimensional scaling (MDS) plot showing the distinction of psoriatic cases into two groups, PP01 (red) and PP02 (black), as obtained after RF clustering and classification. [file 1471-2164-13-472-S3.jpeg]

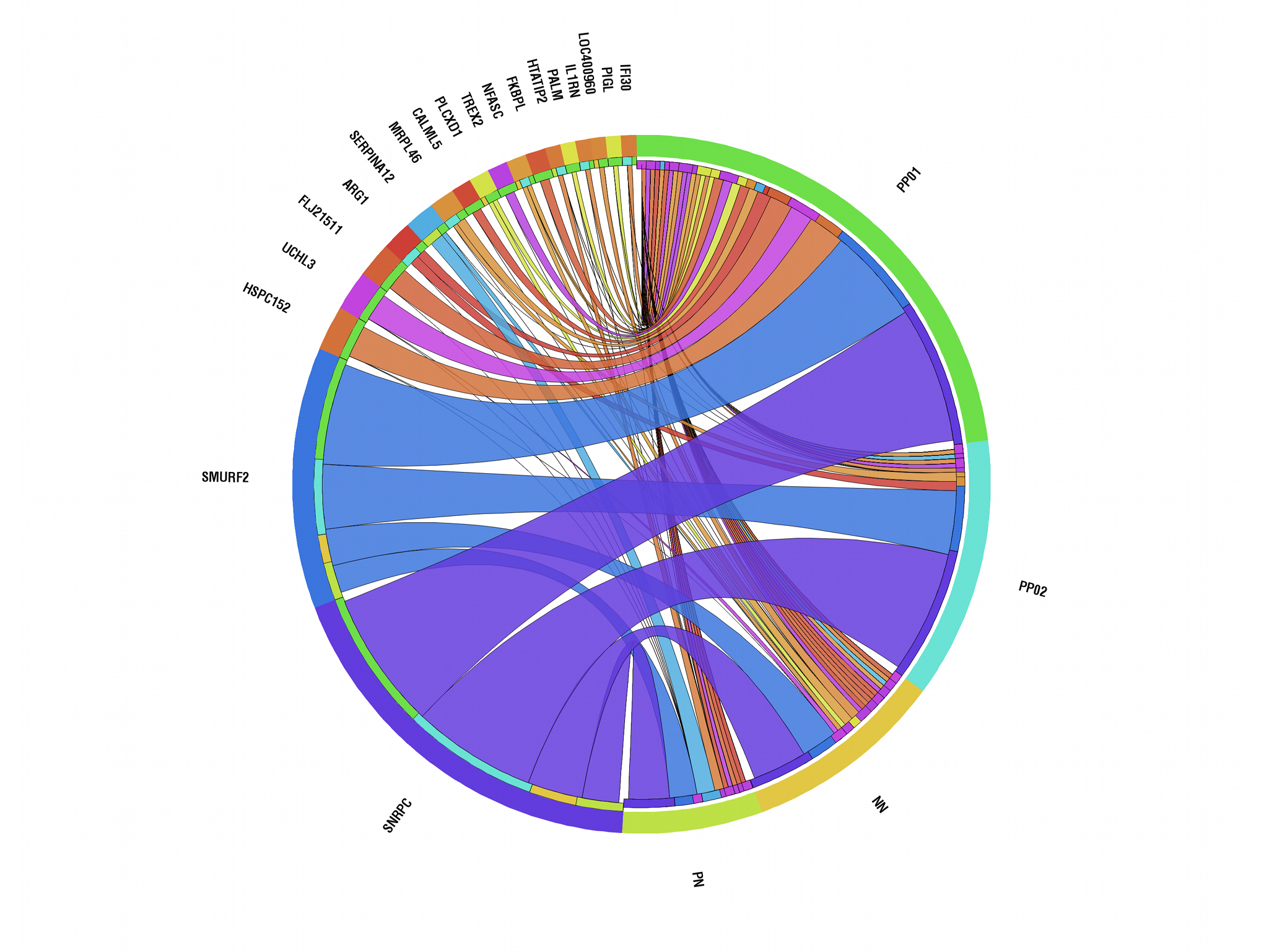

Supplement: Additional File 5 — Genes identified as most informative after classification of skin disease phenotypes. Gini Index (GI) was used as variable importance measure and was estimated for each gene per group from random forest classification, so as to prioritise genes in terms of their ability to discriminate distinct molecular patterns. After training of the random forest classifier, GI is derived for each gene across all trees and the ranking of genes with GI > = 0.02 is shown here for each skin group. [file 1471-2164-13-472-S5.jpeg]

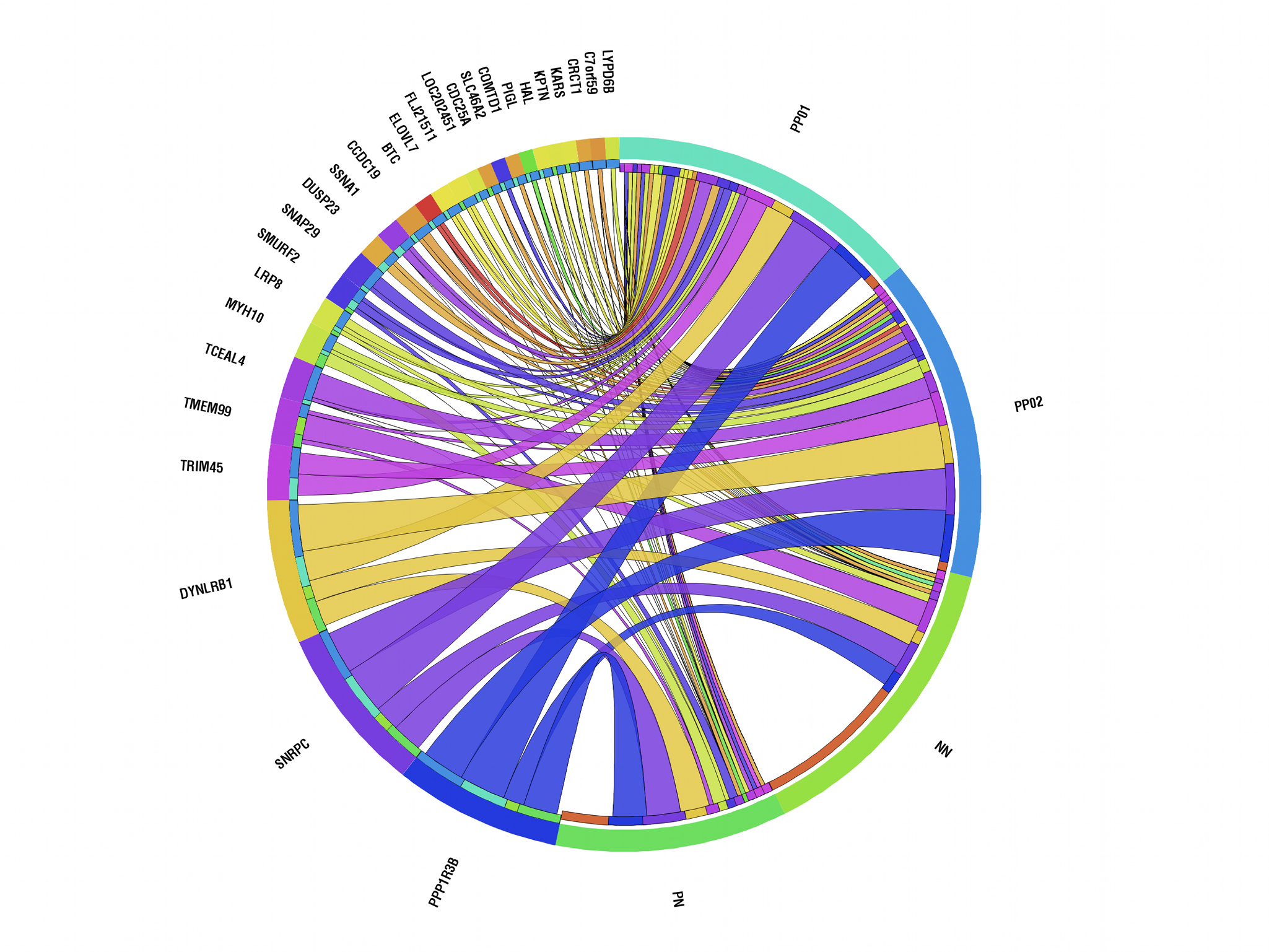

Supplement: Additional File 7 — A multidimensional scaling plot of psoriasis datasets from Gudjonnson et al. 2010 [18] (A) and Yao et al. 2008 [36] (B) to illustrate grouping of samples according to random forest clustering. Two distinct psoriatic groups are identified in involved tissue (PP01 green and PP02 purple), while NN and PN samples largely co-localise. Overall, clustering is comparable to GAIN data that is shown in figure 4. [file 1471-2164-13-472-S7.jpeg]

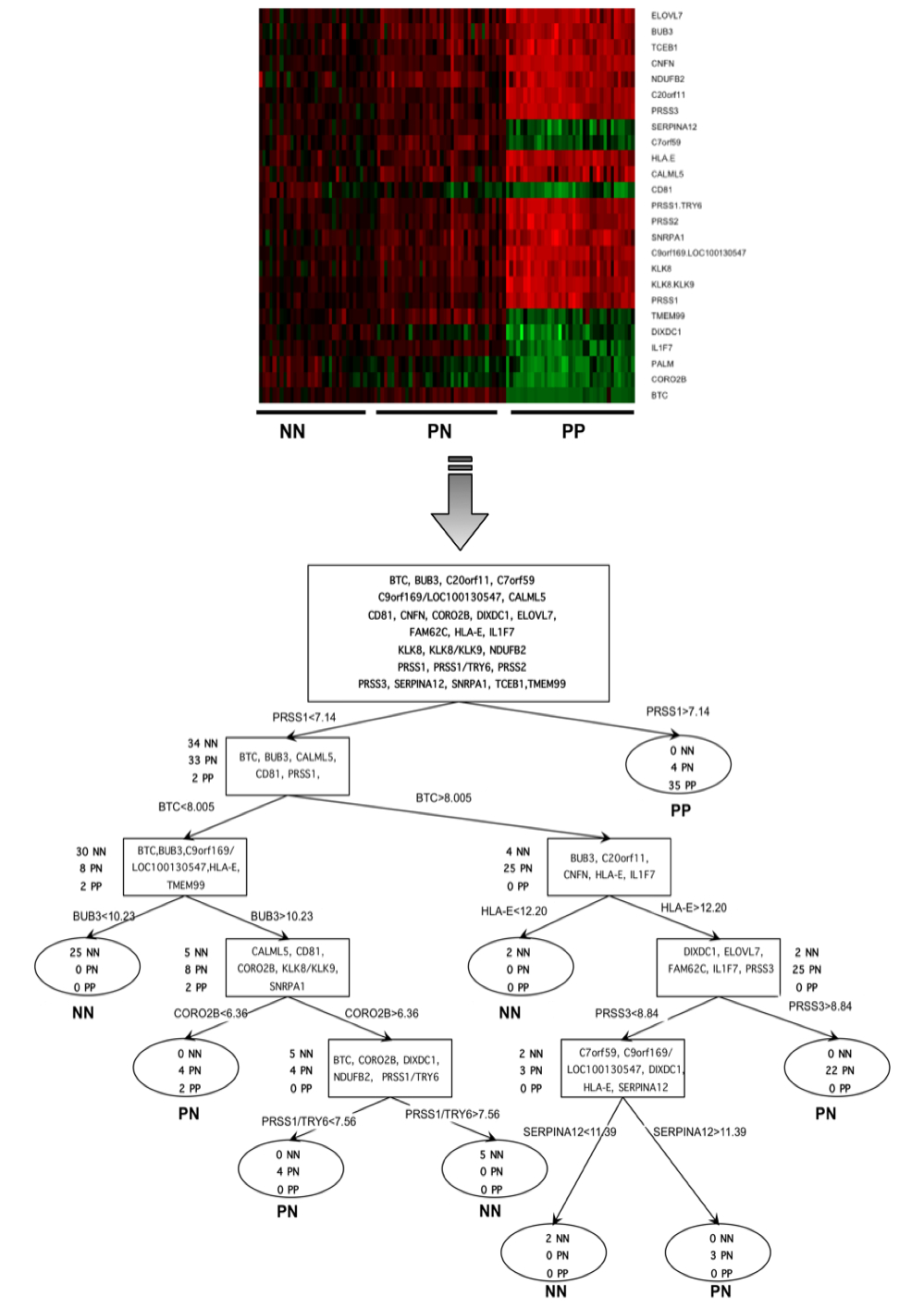

Supplement: Additional File 8 — Graphical representation to illustrate the relationship between 19 highly discriminative genes and disease sub-groups according to Gini Index calculated from decision trees forest in the Gudjonnson dataset. The green band represents the first psoriatic group (PP01), light blue corresponds to the second psoriatic sub-group (PP02), yellow corresponds to healthy individuals (NN) and light green presents the non-lesional cases (PN) and are arranged clockwise followed by purple to orange rectangular bands that represent relevant genes. Genes and skin groups are ordered according to shared pairing links, as described previously. [file 1471-2164-13-472-S8.jpeg]

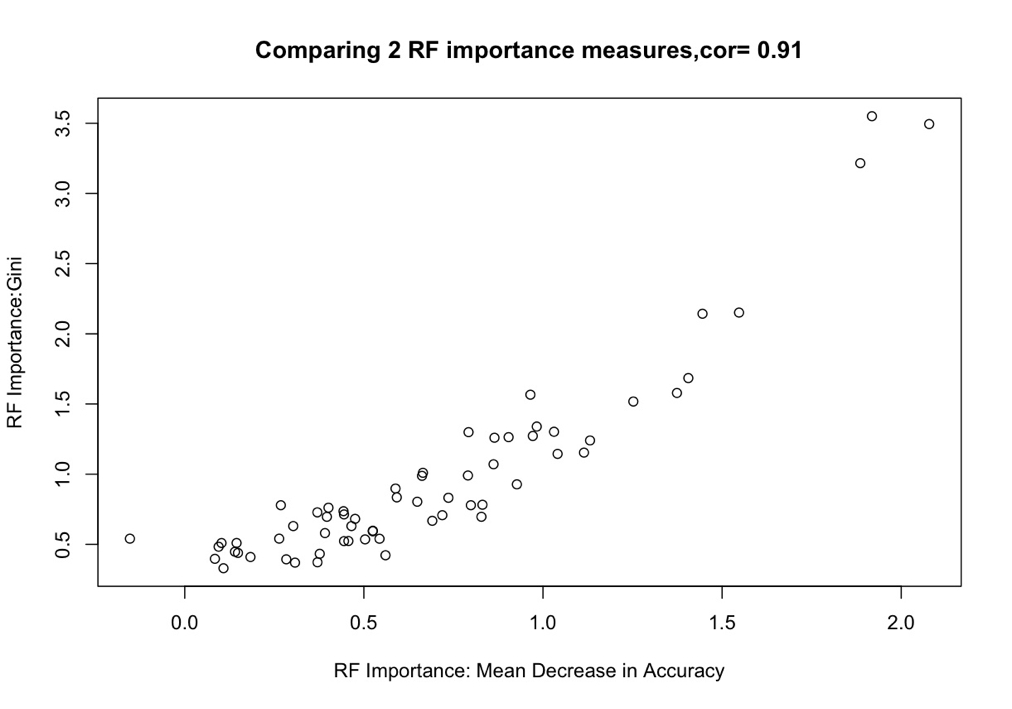

Supplement: Additional File 9 — Graphical representation to illustrate the relationship between 27 highly discriminative genes and disease sub-groups according to Gini Index calculated from RF for the Yao dataset. Light blue to green rectangular bands represent the four skin-types (PP01: light blue, PP02: blue, NN: light- green, PN: green) and are followed by purple to orange rectangular bands representing relevant genes (arranged clockwise). Genes and skin groups are ordered according shared pairing links. An overview of patterns of informative genes for prediction of each disease class can be visualised. [file 1471-2164-13-472-S9.jpeg]

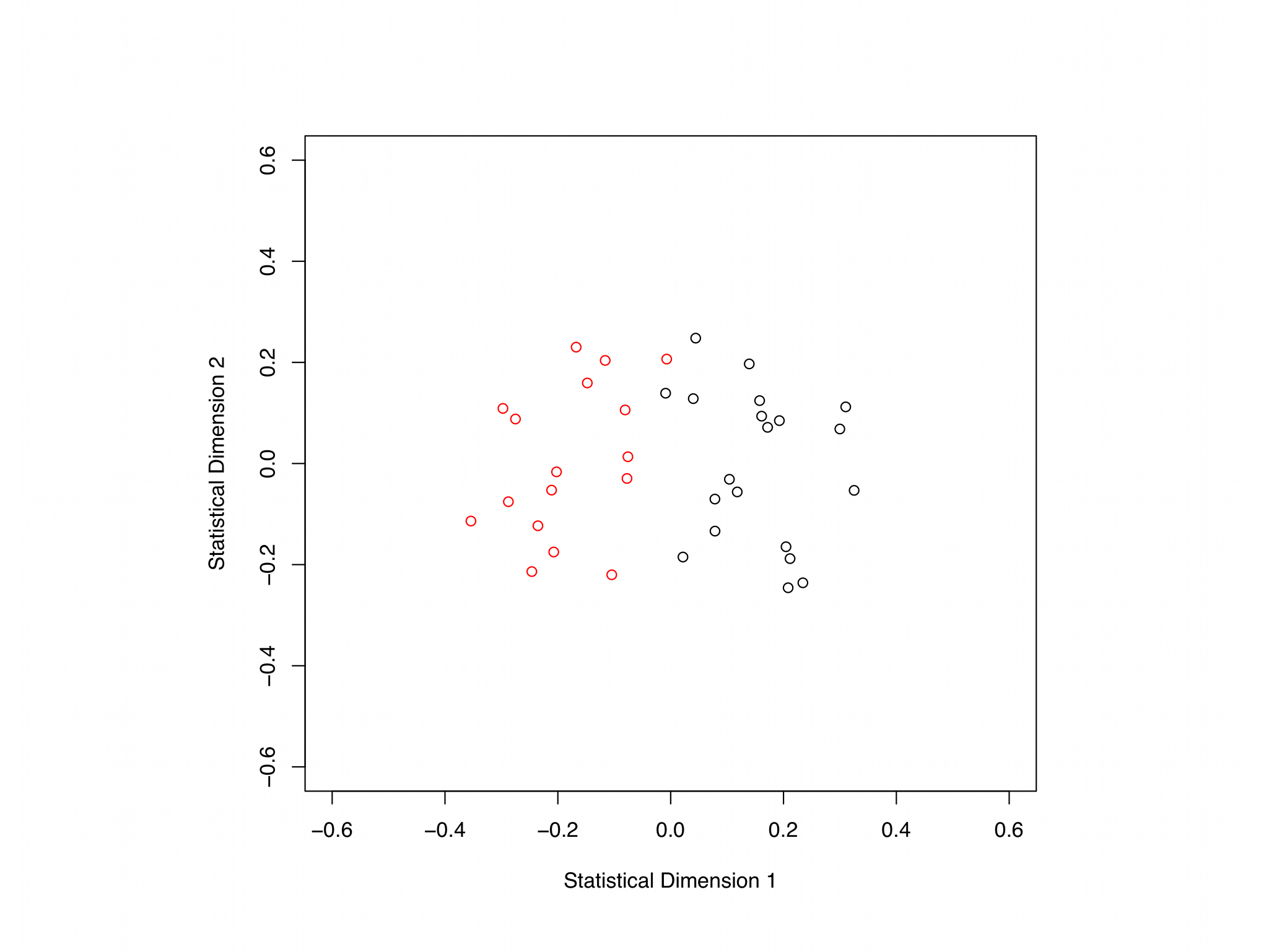

Supplement: Additional File 12 — Example of a decision tree for classification of tissue samples in appropriate disease classes. Heatmap illustrates expression values for 25 genes across 108 tissue samples and represents part of the heatmap shown in figure 2. A decision tree is a tree-like structure to relate gene expression measurements to sample phenotype class, with a view to deriving a predictive model. Nodes (rectangles) in the tree represent a test on gene expressions to derive a decision on a sample’s class, edges (arrows) indicate the expression level of the variable that can best distinguish the samples and leaves (or terminal nodes - circles) represent class predictions. The path from root to each terminal node equates to a list of conditions in the form of gene expression rules that can relate tissue samples to disease phenotype class. [file 1471-2164-13-472-S12.jpeg]

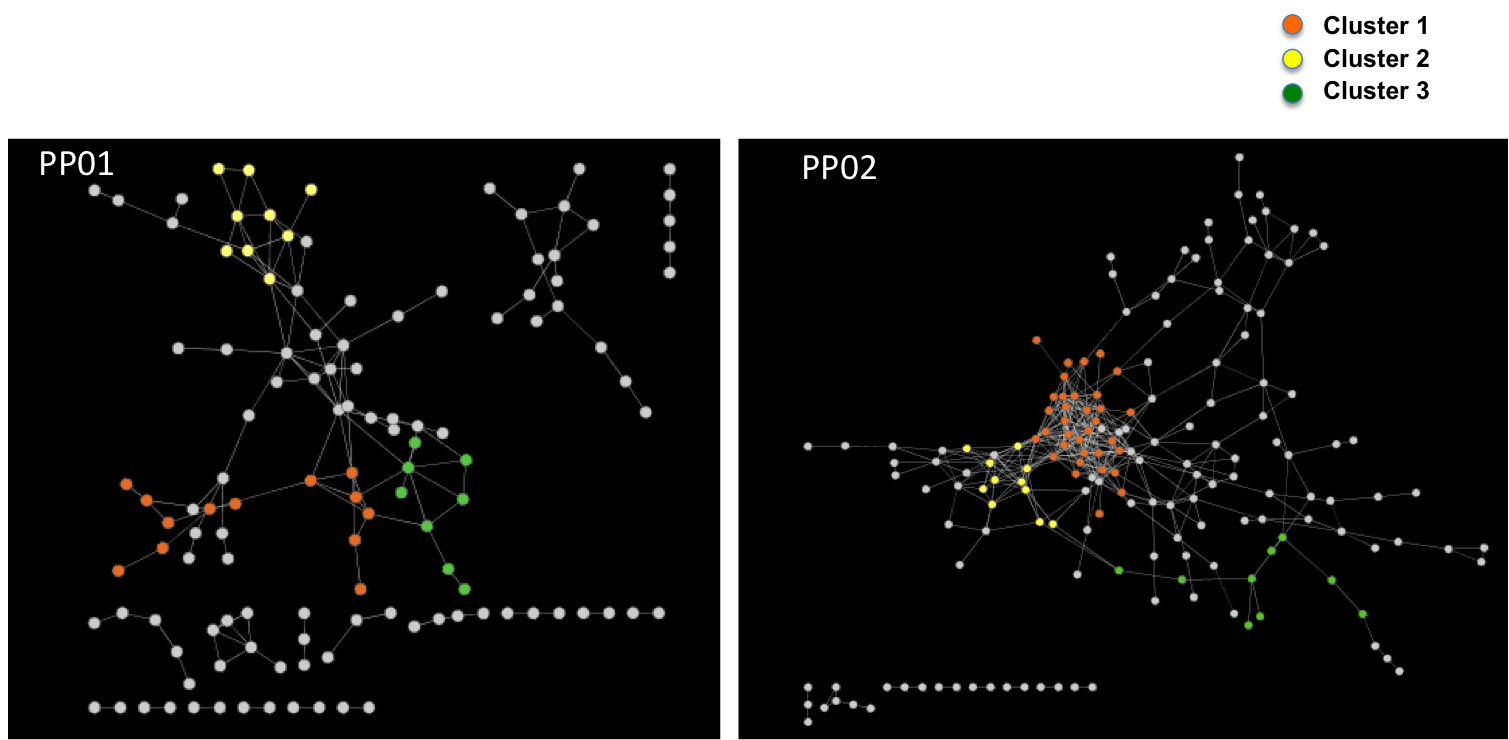

Supplement: Additional File 13 — Correlation between the two variable importance measures of gene selection, Gini Index and mean decrease in accuracy. [file 1471-2164-13-472-S13.jpeg]
